# CD40 Agonist Monoclonal Antibody-Mediated Hepatitis in TNF-Receptor 1 Gene Knockout Mice

**DOI:** 10.3390/biomedicines9080863

**Published:** 2021-07-22

**Authors:** Oksana Raabe, Thomas Birchler, Hubert Rehrauer, Elisabeth Eppler

**Affiliations:** 1 Department of Biomedicine, University of Basel, 4056 Basel, Switzerland; oksana.raabe@unibas.ch; 2 Institute of Experimental Immunology, University of Zurich, 8057 Zürich, Switzerland; thomas.birchler@abbvie.com; 3 Functional Genomics Center Zurich (FGCZ), ETH Zürich, University of Zurich, 8057 Zürich, Switzerland; hubert.rehrauer@fgcz.ethz.ch; 4 Institute of Anatomy, University of Zürich, 8057 Zürich, Switzerland; 5 Institute of Anatomy, University of Bern, 3012 Bern, Switzerland

**Keywords:** TNF-α, TNF receptor 1, CD40 activation, autoimmune hepatitis, mouse model, SBS

## Abstract

Tumor necrosis factor-alpha (TNF-α) plays an important role in liver inflammation. CD40-CD40 ligand (CD40-CD40L) is a key receptor–ligand signaling pair involved in the adaptive immune response and pathogenesis of autoimmune diseases. In mice, CD40 activation leads to sickness behavior syndrome (SBS) comprising weight loss, sleep disruption and depression, which can be blocked by administration of the TNF-inhibitor etanercept. In the present study, we assessed the extent of hepatic inflammation in mice devoid of the TNF-receptor 1 (TNFR1)-mediated signaling pathway. The TNFR1-depleted (TNFR1−/−) adult mice and their wild type littermates were given a single intra-peritoneal injection of CD40 agonist monoclonal antibody (mAb) or rat IgG2a isotope control. As described previously, TNFR1−/− mice were protected from SBS upon CD40 mAb treatment. *Cd40*, *tnf* and *tnfr1* mRNA and Tnf-α peptide were increased in the liver of CD40 mAb-stimulated wild type mice. Serum alanine aminotransferase was elevated in both CD40-activated wild type and TNFR1−/− mice. TNFR1−/− mice showed much less intra-parenchymal infiltrates, hepatocellular necrosis, and perivascular clusters upon CD40 mAb activation than their wild type littermates. A gene expression microarray detected increased activity of metabolic and detoxification pathways and decreased activity of inflammatory pathways. We conclude that immune activation and development of liver inflammation in CD40L interactions depend on TNFR1-mediated signaling pathways and are counteracted by alterations in metabolic pathways.

## 1. Introduction

CD40-CD40L is a key receptor–ligand signaling pair involved in the adaptive immune response [1,2,3,4]. The CD40 immune-activation pathway has been found to play a pivotal role in the host response to infectious pathogens and in the pathogenesis of autoimmune diseases [1,2,5] including autoimmune hepatitis, primary biliary cirrhosis, inflammatory bowel disease, rheumatoid arthritis, and multiple sclerosis [2,6,7]. CD40 and CD40L are widely expressed in various types of cells. Upon activation, CD4+ T lymphocytes and platelets express high levels of CD40L, which stimulates antigen presenting cells (APC), namely dendritic cells, monocytes and macrophages, to produce pro-inflammatory cytokines. CD40L binds to CD40, a member of the tumor necrosis factor receptor (TNFR) superfamily, which is predominantly expressed on B lymphocytes, APC and endothelial cells (5). CD40L-CD40 immune-activation has led to cell migration and production of cytokines such as tumor necrosis factor (TNF)-α and to infiltrates of natural killer cells, B-cells, CD4+ T-cells and APC in various organs, mainly the liver [8,9,10].

Cross-linking of CD40 by monoclonal antibodies activates CD40-expressing cells similar to ligation by CD40L. For instance, activating antibodies to CD40 (CD40 mAb) or soluble CD40L possess therapeutic potential [4,5,7], for example in the treatment of lymphoproliferative malignancies such as non-Hodgkin lymphoma [11], chronic lymphocytic leukemia [12], diffuse large B-cell lymphoma [13,14] and advanced solid tumors [15,16,17]. Nevertheless, their applicability is limited due to the occurrence of chronic fatigue syndrome with signs of fatigue, malaise, decreased appetite, weight loss, reduced social activities, cognitive impairment, depression, and headache [6,18]. For instance, fatigue was reported as common non-hematologic adverse event following all infusion-related reactions [12,13,14], in up to 54% [17] and even 81% of the patients [16].

In mice, experimental autoimmune encephalomyelitis is associated with sickness behavior syndrome (SBS), characterized by weight loss, sleep disruption and depression and has been correlated with pro-inflammatory cytokines [19], while inhibition of CD40 or CD40L rendered mice resistant to experimental autoimmune disorders [2,6]. Since a suitable animal model had not been available for the translational study of the pathophysiology of SBS in autoimmune diseases, a mouse model was established by our group based on a stimulatory CD40 mAb [20]. Intraperitoneal injection of CD40 mAb induced 2–3 day SBS in the form of weight loss, decreased motor activity and sleep alterations [20,21]. Further, splenomegaly attributed to myeloid cell hyperplasia and B-cell expansion was observed [20,22]. CD40 mAb-induced SBS could be prevented in mice co-injected with the TNF blocker etanercept [21]. Furthermore, mice deficient in TNFR1, which binds TNF and lymphotoxin α, were almost completely protected from CD40 mAb-induced loss of locomotor activity and body weight, but not from splenomegaly. Since this protection was not observed for LPS-induced effects, CD40 ligation and LPS stimulation led to SBS via different pathways, and TNFR1 plays a critical role in the CD40-mediated pathway only [20].

TNF-α is also involved in the pathophysiology of hepatitis [23], also upon CD40 activation [24,25], including our own SBS model [26]. In these studies, acute liver toxicity following CD40 mAb injection was characterized by elevated serum levels of transaminases [24,25,26], and ferritin [25], which were abolished in line-selectively depleted CD40-, IFN-γ-, or TNFR1/2-knockout mice [25]. In the latter study, administration of the agonistic anti-CD40 mAb was linked to acute cytokine release syndrome. The concomitant liver damage was considered resembling the secondary macrophage activation syndrome in some patients [25].

Nevertheless, anti-CD40 mAb-activation has not been applied so far to systematically address the CD40 mAb-mediated hepatitis in mice devoid of the TNFR1 signaling pathway in comparison with their wild type (wt) littermates. In this study, we report the extent of tissue inflammation and metabolic alterations during CD40-mediated hepatitis as related to the TNF-α pathway in wt and TNFR1−/− mice in our established model of SBS [20,21,22,26].

## 2. Materials and Methods

### 2.1. TNFR1−/− Mice and Induction of CD40 mAb-Mediated Hepatitis

The present study expands a data set on CD40 mAb-induced SBS [20,21,26] to analyze the observed CD40 mAb-induced hepatitis. The Tnfrsf1atm1Blt termed TNFR1−/− mice were originally kindly provided by Prof. Mathias Heikenwälder, University Hospital Zurich, Switzerland. To induce SBS, the TNFR1−/− adult male C57BL/6J mice and their wt littermates, aged 10–14 weeks (Janvier Labs, Le Genest-Saint-Isle, France) were treated by intra-peritoneal injection with 200 μL PBS, containing either CD40 mAb at a dose of 10 mg/kg or IgG2a control Ab (both from Bio X Cell, West Lebanon, NH, USA) as described [20]. The CD40 mAb is a rat IgG2a mAb (clone FGK4.5), which binds mouse CD40 and activates this receptor molecule [27]. In vivo experiments were conducted at the University of Zurich under license 170/2012 according to the regulations of the Veterinary Office, Canton Zurich, Switzerland.

### 2.2. Experimental Protocol and Tissue Sampling

The experiments were performed as described [20,21,26]. Mice were maintained on a reversed 12:12 h light–dark cycle (lights off at 07:00 h) in an individually-ventilated 2 L type cage system with temperature at 20–22 °C and humidity at 50–60%, which contained sawdust and a sleeping igloo. Food (Complete pellet, Provimi, Kliba Ltd., Kaiseraugst, Switzerland) and water were continuously available. Body weight and weight of pellets eaten as a marker of food consumption were recorded at 14:00 h daily. Mice were handled on each of three days prior to the experiment. As described previously [20,21,26], naïve mice were injected once at day 0 at 18:45 h. On day two, five CD40 mAb- and three IgG2a-injected TNFR1−/− and wt mice, respectively, were sacrificed and tissues sampled. In detail, blood was harvested by cardiac puncture and allowed to clot at 4 °C. For real-time PCR, gene array and total protein extraction, three animals per group were transcardially perfused with ice-cold PBS, and cerebellum, cortex, frontal cortex, hypothalamus, liver, kidney, spleen, thymus and intestine tissue pieces were sampled, immediately snap-frozen in liquid nitrogen and stored at −80 °C until use. For histological analysis, two liver tissue samples of different regions were collected from three CD40 mAb and two IgG2a-treated TNFR1−/− and wt mice, respectively, and fixated overnight at 4° by immersion in 4% formaldehyde buffered in PBS.

### 2.3. Real Time PCR

Gene expression was analyzed as described previously [21,28]. In brief, RNA was extracted by homogenization of the organ in peqGOLD RNA pure (PeqLab, Erlangen, Germany) according to the manufacturer’s instructions. Isolated RNA was DNAse-digested and purified with spin columns NucleoSpin RNA II (Macherey-Nagel, Düren, Germany). Then, cDNA was synthesized using random hexamers and M-MuLV reverse transcriptase (Applied Biosystems, Rotkreuz, Switzerland) as described [21,28,29]: The cDNA equivalent to 20 ng of total RNA was PCR-amplified in an ABI PRISM HT7900 detection system using the TaqMan Universal PCR Master Mix and pre-developed mouse specific TaqMan assays (Applied Biosystems): *tnf-α* (4331182), *tnfr1* (4351370), *cd40*, *18srRNA* (4310893E). All samples were run in duplicate and the results were normalized to *18srRNA* as described [20,21,29]. Relative mRNA levels of treated and control-treated groups were calculated as n-fold expression (mean ΔCT values ± SEM, whereby ΔCT is calculated as the threshold cycle (CT) of the gene of interest—CT of the internal control).

### 2.4. Microarray

Gene expression profiling of liver tissue samples was performed using Agilent’s Whole Mouse Genome Oligo—4X44K microarray (Agilent Technologies, Basel, Switzerland). Images were quantified with the Agilent Feature Extraction Software. Normalization and differential expression were computed using R/Bioconductor. Specifically, the data were quantile normalized. Pathway analysis was computed using the gene set enrichment analysis (GSEA) implemented in the Bioconductor (https://www.bioconductor.org (accessed on 14 July 2021)) package clusterProfiler [30,31].

### 2.5. Serum Analyses

Serum was collected by centrifugation and stored at −20 °C until use. Serum levels of the liver enzyme alanine aminotransferase (sALT), blood urea nitrogen (BUN) and glucose (GLU) were measured by an Abaxis Piccolo Xpress reader (Sysmex Digitana AG, Horgen, Switzerland) using piccolo panel plus assay discs for liver and metabolic markers following the instructions by the manufacturer (Sysmex Digitana AG, Horgen, Switzerland).

### 2.6. Liver Tissue TNF-α ELISA

For total protein extraction and TNF-α measurement, a modified protocol was applied as described [21]. In brief, liver tissue samples were homogenized with a QIAGEN Tissue Lyser II (Qiagen, Venlo, The Netherlands) for 2.5 min/25 Hz in extraction buffer containing 20 mM TrisHCl, 0.15 M NaCl, 2 mM EDTA, 1 mM EGTA, and a protease inhibitor cocktail (Sigma, Buchs, Switzerland). Samples were centrifuged at 1000× *g* for 10 min at 4 °C. Thereafter, the supernatants were removed and centrifuged a second time at 16,000× *g* for 120 min at 4 °C. The total protein concentration content was quantified with a coomassie dye-binding Bradford assay (Bio-Rad, Cressier, Switzerland). The TNF-α content was measured by a Luminex-based system using a mouse specific cytokines Bio-Plex Pro™ assay as described previously [21], following the standard protocol of the manufacturer (Bio-Rad).

### 2.7. Histological and Quantitative Analysis of Liver Tissue

The formaldehyde-fixated specimens were dehydrated in an ascending series of ethanol and routinely embedded in Paraplast Plus (Carl Roth GmbH, Karlsruhe, Germany) at 58 °C. From each tissue sample, 750 μm were cut at consecutive sections of 5 µm, mounted on Super Frost Plus slides (Menzel-Gläser, Braunschweig, Germany) and dried overnight at 42 °C. After dewaxing in Xylol (Fluka, Buchs, Switzerland), sections were rehydrated in a descending series of ethanol (100%, 96%, 70%) and routinely HE stained. In brief, slides were immersed in Mayer’s hematoxylin for 10 min, briefly treated with HCl solution, rinsed in tap water for 10 min and immersed in eosin for another 10 min. After another washing step, differentiation in HCl ethanol was performed followed by rinsing in tap water. Slides were dehydrated in 100% ethanol and Xylol and covered with Eukitt (Merck, Zug, Switzerland). In order to avoid double-counting of the cells, every 20th section was inspected, so that a distance of 100 μm was achieved between the individual sections. Quantitative analysis of tissue inflammation was performed by cell-counting on six different visual fields with regard to hepatic intra-parenchymal infiltrates, perivascular clusters (>100 cells), necrosis infiltrate and total immune cell counts. Microscopic analysis and image acquisition were performed using a Zeiss Axioscope and Axiovision software (Zeiss, Jena, Germany).

### 2.8. Statistical Analysis

Statistical analysis of serum levels and liver histology was performed with an Excel© 2013 software, version 15.0 (Microsoft Corp., Redmond, WA, USA), SPSS (version 20, SPSS Inc., Chicago, IL, USA) and Graph Pad Prism 6.04 (GraphPad Software Inc., La Jolla, CA, USA). Analysis of variance (ANOVA) was conducted with a between-subject factor of treatment (IgG2a, CD40Ab). Post hoc testing was conducted using the Bonferroni test or Tukey’s test. A Kruskal–Wallis test was applied for the histological analyses. Serum data were analyzed by unpaired *t*-test. A two tailed *p*-value < 0.05 was considered statistically significant and exact *p*-values are presented. All quantitative data are presented as mean and standard error of the mean (S.E.M.). Microarray data were quantile normalized and differential expression was computed using *t*-test.

## 3. Results

### 3.1. CD40 mAB Induced Sickness Effects in wt and TNFR1−/− Mice

Similar to previous findings from our group [20,21,22,26], CD40 mAb treatment exhibited sickness effects in wt mice, such as reduced locomotor activity with prolonged resting periods and weight loss, while there was no such effect in IgG2a mice. Additionally, as described in [20,21], TNFR1−/− mice were protected from SBS upon CD40 mAb treatment, but similar to their wt littermates developed splenomegaly (data not shown).

### 3.2. Tissue Distribution of tnf, tnfr1, and cd40

**Figure 1:**
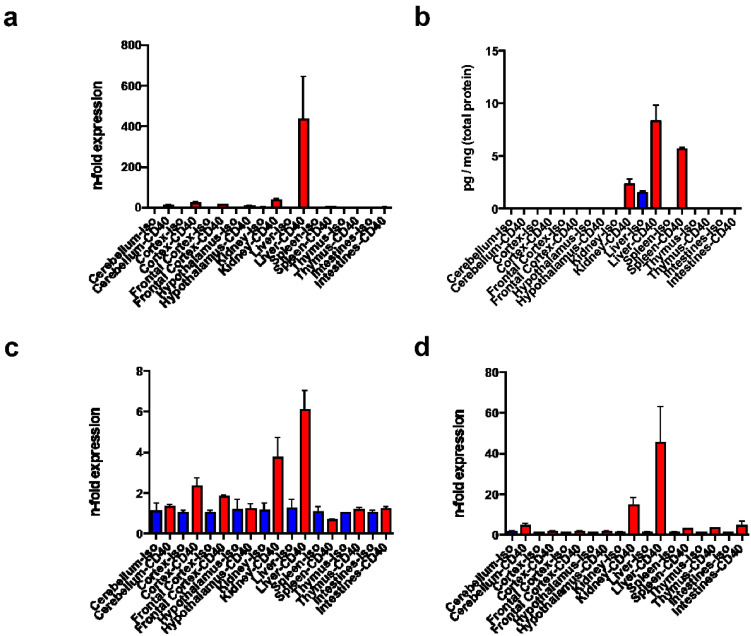
Tissue distribution of *tnf*, *tnfr1* and *cd40* gene expressions (**a**,**c**,**d**) and TNF-α protein levels (**b**) in cerebellum, cortex, frontal cortex, hypothalamus, kidney, liver, spleen, thymus and intestine (X-axis) of CD40 mAb (-CD40, red columns) and control-treated (-Iso, blue columns) wt mice. (**a**,**c**,**d**) Real time PCR data for *tnf* (**a**), *tnfr1* (**c**) and *cd40* (**d**) gene expressions as n-fold variations (Y-axis) calculated as mean ΔCT values normalized to *18srRNA* as internal control. (**b**) ELISA assay for TNF-α peptide content (Y-axis: pg/mg of total protein, mean values). Error bars: S.E.M.

Gene expressions of *tnf*, *tnfr1*, and *cd40* were analyzed in CD40- and isotype-treated wt mice. The reference gene *18srRNA* was stable across the groups as described [20,21,29]. Increased gene expression of *tnf* was measured in liver of CD40 mAb stimulated mice (Figure 1a). Additionally, lso TNF-α peptide was elevated in the liver, followed by spleen and kidney, upon CD40 mAb activation (Figure 1b). Furthermore, *tnfr1* and *cd40* gene expressions were increased in liver, and to a lesser extent in kidney upon CD40 mAb activation, whilst baseline expressions were observed in other tissues independent of CD40 mAb treatment (Figure 1c,d).

### 3.3. Gene Expression Microarray Analysis of Liver Tissue

**Figure 2:**
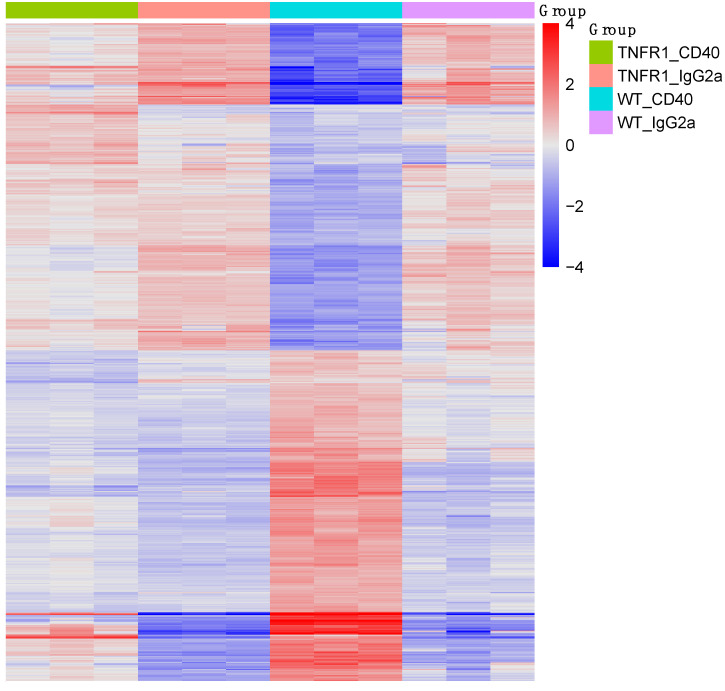
Heat map of the differential expression array of over-expressed (red) and under-expressed (blue) genes (Table 1) in TNFR1−/− mice treated with CD40 mAb (green) relative to wt mice treated with CD40 mAb (turquoise). Orange color depicts TNFR1−/− mice, and violet color wt mice, treated with the control IgG2a, respectively.

We performed differential expression analysis using Agilent gene expression arrays and compared the expression profiles of TNFR1−/− mice treated with CD40 mAb relative to wt mice treated with CD40 mAb (Figure 2). Using a *p*-value threshold of 0.01 and fold-change threshold of 2.0, in total 974 genes were described as significantly changed. The false discovery rate for these genes was 0.038. Genes with higher expression in CD40 mAb-stimulated, TNFR1−/− mice were associated with metabolic and detoxification pathways, while inflammation related pathways like cytokine interaction, as well as NF-kappa B, IL-17, and TNF signaling were associated with under-expressed genes (Table 1).

### 3.4. Serum Levels of sALT, BUN and GLU

Confirming previous findings from our group [26], sALT concentration as a marker of hepatocyte destruction was significantly elevated in wt mice after CD40 mAb-activation to 14.3 fold of the mean (*p* = 0.0003) as compared to control-injected mice. The sALT was also significantly elevated (*p* = 0.0012) by three-fold of the mean in CD40 mAb-activated wt mice as compared to TNFR−/− animals, which, however, also showed significantly elevated sALT levels by four-fold of the mean (*p* = 0.0002) as compared to control injected TNFR−/− mice. In both, wt and TNFR−/− mice, no significant changes in BUN and GLU serum levels as metabolic markers were measured after CD40 mAb treatment (data not shown).

### 3.5. Histological Analysis of Hepatic Inflammation

**Figure 3:**
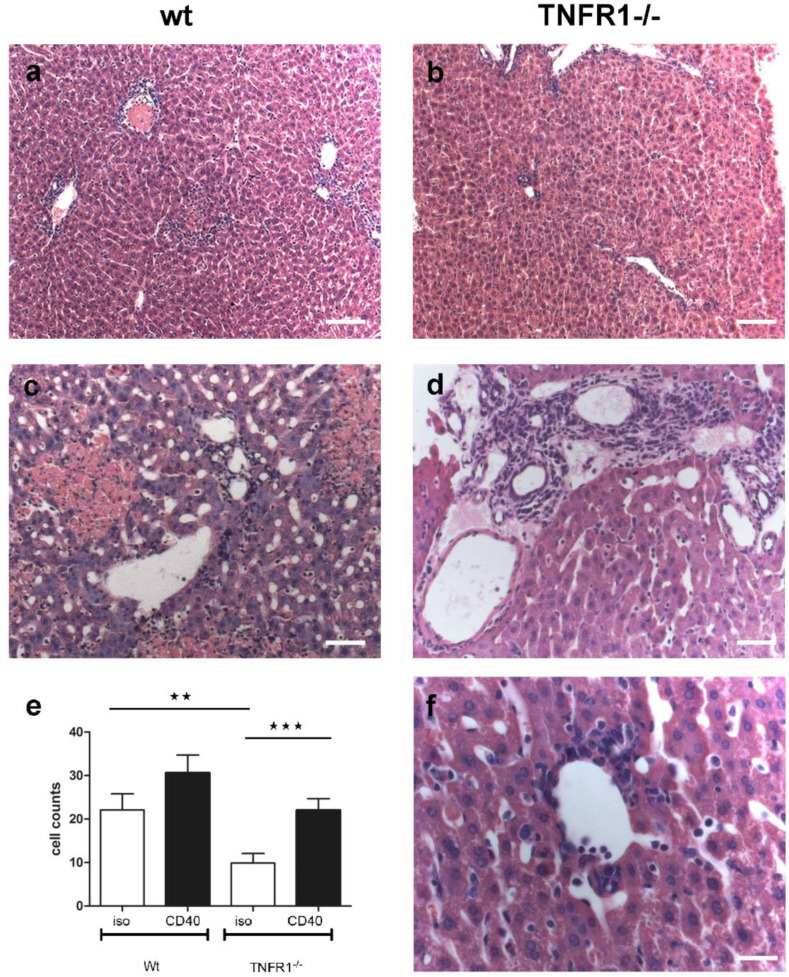
Light microscopic analysis of hepatic tissue sections of wt (**a**,**c**) and TNFR1−/− mice (**b**,**d**,**f**). (**a**–**d**,**f**) HE-stained liver tissue from CD40 mAb activated (**a**,**c**) wt and (**b**,**d**,**f**) TNFR1−/− mice. Bars: (**a**,**b**): 500 µm, (**c**,**d**): 200 µm. (**f**): 100 μm. (**e**) Semiquantitative analysis of cell counts on the HE-stained sections of isotype (white columns) and CD40 mAb-treated (black columns) mice. ** *p* = 0.006, *** *p* = 0.0008. Error bars: S.E.M.

Histological analysis of liver tissues revealed in wt mice an infiltration of leukocytes around the interlobular tract and in the central veins upon CD40 mAb activation (Figure 3a). Additionally, pronounced hepatocellular necrosis and inflammatory cell accumulation (Figure 3c) were observed with infiltration of leukocytes in the liver parenchyma, and around the interlobular bile-duct and blood vessels. Nevertheless, no significant (*p* = 0.07) difference in total cell count (Figure 3e) was observed between CD40-treated wt mice (mean 30.6 ± 4.1 cells, range: 7.0–67.0 cells, median 30.5) as compared to IgG2a-injection (mean 22.1 ± 3.7 cells, range: 6.0–47.0 cells, median 20.0 cells). These higher immune cell counts (*p* = 0.006, Figure 3e) in isotype-treated wt mice compared to TNFR1−/− mice (Figure 3e) were mainly derived from significantly (*p* = 0.0004) more perivascular clusters (mean 5.3 vs. 3.8).

Different to wt, upon CD40 ligation the TNFR1−/− mice preserved the cellular organization of normal mouse liver (Figure 3b). Although typical inflammatory signs were also observed in TNFR−/− mice upon CD40 activation (Figure 3d), they showed significantly less (*p* = 7.8 × 10^−9^) intra-parenchymal infiltrates (average 2.1 vs. 5.1) and almost no (*p* = 2.2 × 10^−9^) necrosis (average 0.2 vs. 4.4) as compared to their wt littermates (Figure 3c,d). In some TNFR1−/− mice treated with CD40 mAb, moderate immune cell accumulations mainly in the portal areas (Figure 3d) and in some central venules were detected (Figure 3f), so that in the CD40-treated TNFR1−/− mice on average significantly more (*p* = 0.0008) cells (22.0 ± 2.7 cells, range: 5.0–40.0 cells, median: 22.0 cells) were counted (Figure 3e) than after control treatment (9.9 ± 2.2 cells, range: 1.0–21.0 cells, median: 10.0 cells).

## 4. Discussion

The observed sickness effects on wt mice, which were similar to previous findings from our group [20,21,22,26], assured that CD40 mAb treatment was successfully performed while sickness effects were not observed in control-injected mice and in TNFR1−/− mice as described [20]. Thus, the present study was comparable to previous findings and could be used to explore the effects of CD40 activation on liver inflammation in our SBS mouse model [21,26], in TNFR−/− mice and their wt littermates.

Investigation of baseline and CD40 mAb-induced tissue distribution of *tnf*, *tnfr1*, and *cd40* revealed increased expression of the *tnf* gene and TNF-α peptide in the liver of CD40 mAb stimulated mice, and to a much lesser extent the *tnf* gene in kidney two days after CD40 mAb injection. Similarly, *tnf* gene expression was elevated nine hours after CD40 mAb injection in kidney, but also in brain and spleen [21], so that the elevated spleen TNF-α peptide in the present study may be either produced earlier or derived from immigrating immune cells and thus contribute to the development of SBS.

In the present study, also *tnfr1* and *cd40* gene expressions were increased upon CD40 mAb stimulation in liver and to a lesser extent in kidney. CD40 is expressed predominantly on endothelial cells, B lymphocytes and APC [5] and in various cell types of the diseased kidney, including epithelial cells, podocytes, and immune cells so that CD40 is considered as important mediator in the regulation of inflammatory and fibrotic kidney processes [32].

Our observed elevations of *cd40* gene expression and TNF-α peptide content in the liver were probably also due to increased accumulation of immune cells, as could be visualized by histological investigations in the present study. For instance, recruitment of immune cells including T and B lymphocytes to the site of injury or inflammation via a low-flow hepatic sinusoidal endothelial vascular bed was observed during hepatic inflammation [9,23,24], whereby hepatic B cell recruitment is mainly macrophage-independent [9]. For instance, in a mouse model of macrophage activation syndrome, liver toxicity upon CD40 mAb ligation was abolished after macrophage depletion or macrophage-selective *cd40* gene deletion [25]. Furthermore, in another murine model of fulminant hepatitis, *cd40* expression was markedly increased in hepatocytes [8], so that our observed increase in *cd40* gene expression was probably also derived from hepatocytes and not from immune cells exclusively.

In anti-CD40 mAb-induced necro-inflammatory liver disease, the importance of TNF-α has been emphasized [9,24,25]. In the present study, we assessed the extent of inflammation and metabolic course of hepatitis in mice devoid of the TNFR1-mediated signaling pathway. The sALT concentration was investigated as a marker of hepatocyte destruction and, confirming previous findings from our group [26], was significantly elevated in CD40-activated wt mice as compared to their control-treated littermates. While this CD40 mAb-provoked increase was significantly less pronounced in the TNFR1−/− mice, the sALT was still significantly higher in CD40 mAb-treated than in isotype-treated TNFR1−/− animals, which is well in accordance with our morphological detection of increased immune cell accumulations also in the TNFR−/− mice.

In the present study, the wt mice revealed necro-inflammatory liver disease upon CD40 activation with pronounced areas of hepatocellular necrosis and inflammatory cell accumulation. Large areas of necrotic liver parenchyma after CD40 administration were similarly observed adjacent to thrombotic vaso-occlusions, which was attributed to acute ischemic liver disease [25].

In contrast, in the present study, upon CD40 activation the TNFR1−/− mice preserved the cellular organization of normal mouse liver, which is similarly arranged to other mammalian species [33]. While in some individuals, moderate immune cell accumulations were observed mainly in the interlobular areas and some central venules, the extent of disease was limited to these inflammatory signs, but almost no necrosis was found. Whilst quantification revealed in TNFR1−/− mice less intra-parenchymal infiltrates, there was, nevertheless, no significant difference in the total cell count upon CD40 between wt and TNFR1−/− mice. The generally fewer immune cells in the TNFR1−/− mice were mainly derived from perivascular clusters, so that a depletion of the TNF receptor has obviously led to a shift in the hepatic immune cell distribution pattern. Interestingly, our data resemble previous observations in lymphotoxin β receptor-depleted mice, such as massive infiltration of lymphocytes around perivascular areas in lungs, liver, pancreas, submandibular glands, fatty tissue of mediastinum, mesentery, cortex of the suprarenal glands, and kidney [34].

The effects in our C57BL/6 wt mice were similar to intra-parenchymal infiltrates, perivascular clusters and necrosis as well as sALT elevation in tumor free C57BL/6 and BALB/c mice treated with 100 μg agonistic CD40 mAb [24]. Interestingly, autoimmune cholangitis was transiently delayed by CD40 activation in a murine model of primary biliary cirrhosis, which expresses a dominant form of transforming growth factor-β receptor type II. In that model, CD40 activation reduced the frequency of effector T cells and natural killer cells within the hepatic parenchyma and T-cell activation in the peripheral blood. Additionally, TNF-α serum levels were lowered in that study [35] in contrast to the elevated TNF-α serum levels in SBS, which could be abolished by reducing TNF receptor-binding by etanercept [21].

Thus, modulating cytokines such as the TNF-α pathway differentially interacts with physiological functions in mice. Immune challenge is an energy-consuming process, which is at a cost to other physiological functions such as metabolism or reproduction or vice versa. This is supported by our gene expression array, where genes with higher expression in CD40 mAb-stimulated TNFR1−/− mice were associated with metabolic and detoxification pathways, while inflammation-related pathways like cytokine interaction, as well as NF-kappa B, IL-17, and TNF signaling were associated with under-expressed genes. While TNFR−/− mice were protected, treatment with anti-CD40 mAb in wt mice acutely induced 2–3 day SBS in the form of weight loss, decreased motor activity and increased NREM sleep, sleep fragmentation and brief awakenings as described [20,21], whereby the reduced locomotor activity was mainly characterized by prolonged resting periods [20]. Elevated TNF-α in vitro interfered with clock gene *dbp* expression in the suprachiasmatic nucleus and may prolong the resting periods in the dark when mice are mostly active [29]. CD40 mAb-induced SBS also included weight loss [20,21], which at first glance would not be expected in a reduced locomotor activity state. However, increased peripheral TNF-α levels led to weight loss in a mouse model of sickness and depression- and anxiety disorder-relevant behavior via different pathways [36]. A previous study from our group suggested that the observed sleep–wake dysregulations in autoimmune diseases may result from CD40-induced TNF-TNFR1 mediated alterations of molecular pathways, which regulate the sleep–wake behavior [21]. Our observed metabolic alterations, which were predominantly up-regulated, while immunological genes were predominantly down-regulated, may contribute to a better understanding of metabolic changes such as weight loss or gain in stressful life phases. For instance, metabolic syndrome was worsened and hepatic inflammation ameliorated upon depletion of CD40 on CD11c+ in a model of dietary non-alcoholic steatohepatitis [37].

Protein–protein interactions of costimulatory or coinhibitory cytokines are being targeted by numerous clinical approaches [38]. Here, we demonstrated that adverse effects in the liver by targeting CD40-CD40L using anti-CD40 mAb were also mediated via TNF signaling.

## 5. Conclusions

We conclude that immune cell infiltration and local liver inflammation in CD40L interactions depend on TNFR1-mediated signaling pathways and are accompanied by alterations in metabolic pathways.

## Figures and Tables

**Table 1:** Top 10 KEGG pathways in CD40 mAb-stimulated TNFR1−/− and wt mice.

Over-Expressed	FDR	Under-Expressed Genes	FDR
Ascorbate and aldarate metabolism	3.52 × 10^−9^	Protein processing in endoplasmic reticulum	3.52 × 10^−9^
Steroid hormone biosynthesis	3.52 × 10^−9^	Ribosome	2.34 × 10^−7^
Retinol metabolism	3.52 × 10^−9^	Cytokine-cytokine receptor interaction	3.38 × 10^−6^
Metabolism of xenobiotics by cytochrome P450	3.52 × 10^−9^	NF-kappa B signaling pathway	3.98 × 10^−6^
Drug metabolism—cytochrome P450	3.52 × 10^−9^	IL-17 signaling pathway	6.53 × 10^−6^
Drug metabolism—other enzymes	3.52 × 10^−9^	TNF signaling pathway	1.67 × 10^−5^
Bile secretion	3.52 × 10^−9^	NOD-like receptor signaling pathway	4.22 × 10^−5^
Chemical carcinogenesis	3.52 × 10^−9^	N-Glycan biosynthesis	2.05 × 10^−4^
Linoleic acid metabolism	1.47 × 10^−8^	Protein export	2.17 × 10^−4^
Olfactory transduction	1.89 × 10^−8^	Viral protein interaction with cytokines and cytokine rec.	2.96 × 10^−4^

Top 10 KEGG pathways identified by gene set enrichment analysis (compare Figure 2) to be associated with over-expressed and under-expressed genes in CD40 mAb-stimulated TNFR1−/− mice as compared to wt littermates. FDR: false discovery rate.

## Data Availability

Data supporting reported results can be obtained from the corresponding author upon request.
